# Automated modeling of protein accumulation at DNA damage sites using qFADD.py

**DOI:** 10.1017/S2633903X22000083

**Published:** 2022-08-30

**Authors:** Samuel Bowerman, Jyothi Mahadevan, Philip Benson, Johannes Rudolph, Karolin Luger

**Affiliations:** 1 Department of Biochemistry, University of Colorado Boulder, Boulder, Colorado, USA; 2 Howard Hughes Medical Institute, University of Colorado Boulder, Boulder, Colorado, USA; 3 Interdisciplinary Quantitative Biology Program, University of Colorado Boulder, Boulder, Colorado, USA

**Keywords:** laser microirradiation, DNA repair, monte carlo simulation

## Abstract

Eukaryotic cells are constantly subject to DNA damage, often with detrimental consequences for the health of the organism. Cells mitigate this DNA damage through a variety of repair pathways involving a diverse and large number of different proteins. To better understand the cellular response to DNA damage, one needs accurate measurements of the accumulation, retention, and dissipation timescales of these repair proteins. Here, we describe an automated implementation of the “quantitation of fluorescence accumulation after DNA damage” method that greatly enhances the analysis and quantitation of the widely used technique known as laser microirradiation, which is used to study the recruitment of DNA repair proteins to sites of DNA damage. This open-source implementation (“qFADD.py”) is available as a stand-alone software package that can be run on laptops or computer clusters. Our implementation includes corrections for nuclear drift, an automated grid search for the model of a best fit, and the ability to model both horizontal striping and speckle experiments. To improve statistical rigor, the grid-search algorithm also includes automated simulation of replicates. As a practical example, we present and discuss the recruitment dynamics of the early responder PARP1 to DNA damage sites.

## 1. Introduction

Living cells are constantly bombarded by DNA damaging agents, from sources both within and outside the cell. This damage can take many forms, such as double-strand DNA breaks, single-strand breaks, or base oxidation events^(1–3)^. If unchecked, the resulting base changes (mutations), deletions, or chromosome fusion and breakage events result in permanent changes in the genome, with often detrimental effects for the cell. To protect from these damage events, cells have evolved a complex array of DNA repair pathways that utilize a wide variety of signaling and repair enzymes, each with varying activities throughout the cell cycle and the DNA damage response^(4)^. While specific pathways counteract particular modes of damage, some proteins can participate in several repair processes, and inhibiting one repair cascade can alter the typically conserved sequential accumulation of signaling and repair proteins^(5)^. Improper regulation of DNA damage sensing and repair proteins is strongly correlated with, and can even result in, several cancers^(6–8)^. To this end, a better understanding of DNA damage pathways and the dynamics of the participating proteins is important to develop better cancer therapeutics, many of which function by introducing excessive DNA damage in rapidly growing cancer cells. As such, suppressing DNA damage repair in these cells will lead to more effective cancer drugs.

Accurate measurement of accumulation kinetics for the many proteins involved in signaling and repairing DNA damage events is necessary to understand the complex interplay within and between the various repair pathways. Laser microirradiation is one popular in vivo technique to study these kinetics due to its ability to track protein dynamics as a direct response to locally induced damage^(9–11)^. In this method, cells are transfected to express one or several fluorescently tagged proteins (depending on microscope capability). A defined region of chromatin is damaged with a short-wavelength (~400 nm) laser and then monitored for time-dependent accumulation of the labeled protein(s) via fluorescence microscopy^(12–14)^. Historically, these data have been fit with such methods as: determining the time required for half of the total accumulation (*t*_1/2_), fitting the time series to a single exponential, and fitting the time series with multiple exponentials^(5,14,15)^. While straightforward to implement, we have previously shown that these methods lack the ability to differentiate between different nuclear shape profiles and therefore suffer from averaging diverse nuclei into a single, representative time series^(13)^. Moreover, as they are not direct measures of diffusion, the output from these analysis methods cannot be directly compared to orthogonal diffusion methods such as FRAP or fluorescence correlation spectroscopy^(9,16,17)^.

**Figure 1.**
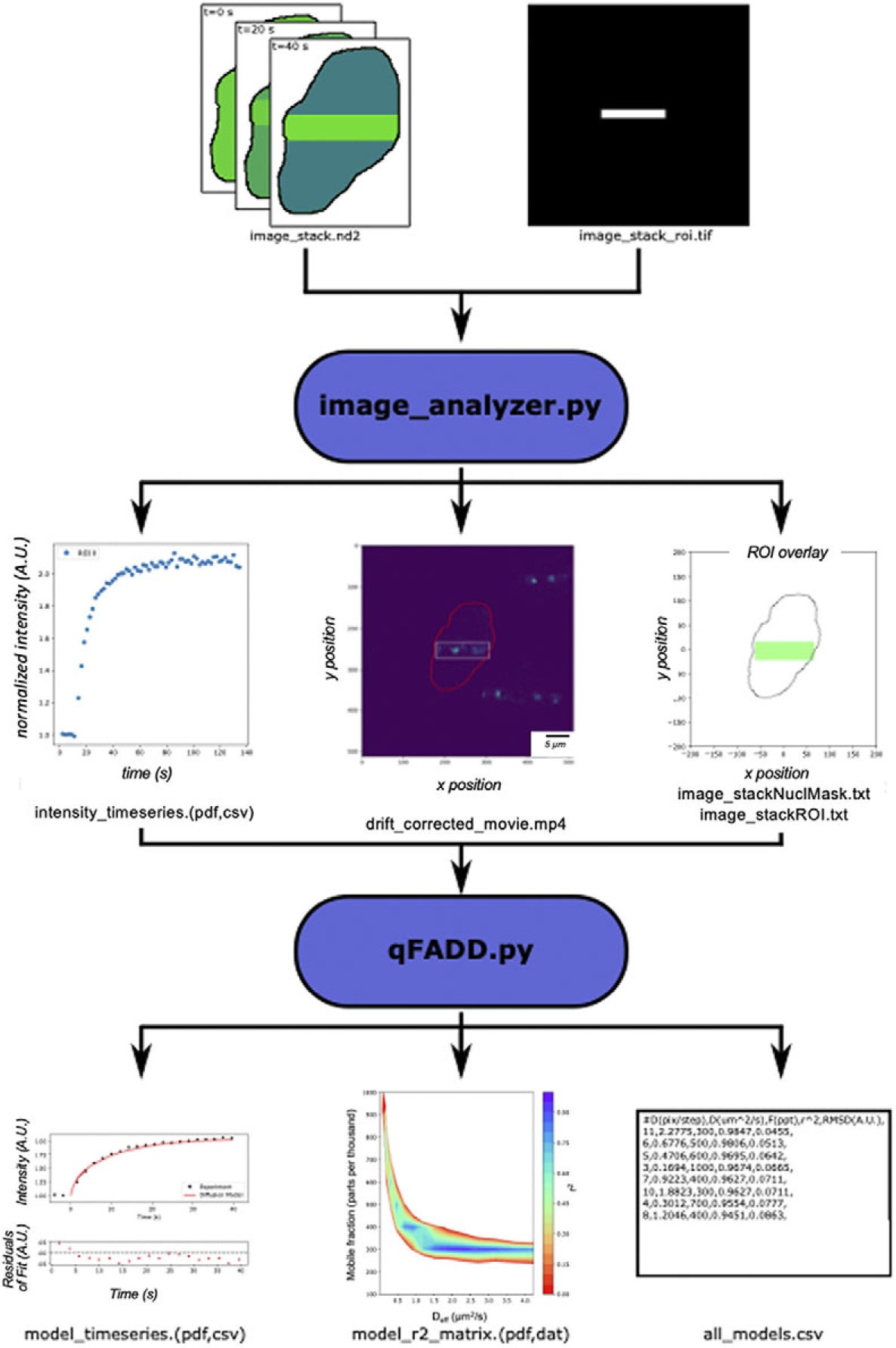
Workflow of the qFADD.py pipeline. Image stacks (snapshots as a function of time) of fluorescently tagged protein accumulation (top left) and a single shape of the region of interest (ROI, top right) are collected by the user on a confocal microscope—here, Nikon/.nd2 files, but all bioformats-readable files are supported. These files are imported into the image_analyzer.py program, which applies motion corrections to the raw image stacks. The output from image_analyzer.py includes the quantitated accumulation time-series data, a drift-corrected movie, and a single trace of the nuclear envelope with the ROI inside the nucleus for input to the qFADD.py modeling program (middle row of boxes). An example ROI for highly damaged “horizontal striping” analysis mode is shown, but qFADD.py is also capable of modeling in “speckle” analysis mode for more localized recruitment events. qFADD.py then conducts the grid-search fitting on a range of *D_eff_* and *F* (mobile fraction) values defined by the user to identify the best-fit model. A plot of the model versus experiment fit is generated, and all sampled models throughout the grid are sorted by fit quality in a human-readable text file (all_models.csv; bottom row). A visual comparison between models is also provided in a heatmap-style plot of model qualities for rapid assessment of overall performance of the defined grid.

To address these limitations, we recently developed a novel method for the analysis of data from laser microirradiation experiments, namely “quantitation of fluorescence accumulation after DNA damage” (Q-FADD)^(13)^. Q-FADD models protein motion as a free-diffusion process and compares the simulated curves to the experimental data in the analysis of laser microirradiation data, which then allows the results to be compared to other diffusion-based methods. The accumulation of protein at sites of DNA damage is described by two variables: *D*_*eff*_, the effective diffusion coefficient, and *F*, the mobile fraction of proteins. These two values are varied to generate an optimal fit to the experimental data. Because shape and size are explicitly represented in the fitting process for each nucleus, Q-FADD is a more appropriate method compared with averaging multiple nuclei and calculating average *t*_1/2_s or exponential fits. We originally developed Q-FADD through a combination of MATLAB and Mathematica notebooks that are not readily accessible to many cell biologists. To make our analytical method more widely available, we developed “qFADD.py” (Figure 1)—an open-source Python implementation of the Q-FADD workflow that exhibits several significant improvements and updates compared to the original method. Other than a more user-friendly application, we have implemented a grid-search algorithm that automatically generates and compares multiple simulated model replicates over a wide range of parameter combinations (*D*_*eff*_ and *F*). This improvement reduces the trial-and-error approach in the original version and the potential for user bias, thereby increasing the statistical certainty of reported best-fit models. Additionally, the new companion program (“image_analyzer.py”) accounts for nuclear drifting, thereby reducing the number of microirradiation datasets that may need to be discarded from analysis or give faulty outputs if not discarded. Laser microirradiation data of the PARP1 signaling protein are presented as examples for the pipeline, and the interpretation of qFADD.py outputs are discussed.

## 2. Methods

Both qFADD.py and associated preprocessing program, image_analyzer.py, are written in Python (v3.6.5), and the code is freely available on GitHub (https://github.com/Luger-Lab/Q-FADD). The GitHub site includes three folders: Documentation, Example_Usage, and src. The Documentation folder has installation instructions (install.md) and a detailed set of instructions for using the graphical user interface (GUI; qFADD_gui_documentation.pdf). The Example_Usage folder has sample raw data as well as a folder of precomputed results for reference. The src folder contains source .ui and .py files. The stand-alone GUIs (“qFADD_gui.py” and “image_analyzer_gui.py”) were constructed with PySide2 and Qt (v5)^(18)^. Parallel operation is achieved through the mpi4py module, which is constructed on top of the openMPI library^(19,20)^, and tasks can either be run locally or submitted to an SLURM queuing system^(21)^. Below we describe both the preprocessing and the fitting procedure.

### 2.1. Extraction of accumulation time-series data

In the laser microirradiation method, fluorescently tagged proteins are expressed in a cell line of choice. Then, DNA damage is induced in a region of interest (ROI) within the nucleus using a highly focused laser beam, and an accumulation of the fluorescently labeled protein(s) in the damaged ROI is tracked. One common approach for inducing damage is through “horizontal striping,” where cells receive a large amount of damage that spans the full width of the nucleus. Alternatively, the beam can be focused to a narrow ROI, thereby inducing damage in a highly localized area (hereby referred to as the “speckle” method). The raw data from these measurements are typically a time-lapsed image stack of the accumulation process (i.e., individual snapshots of the nucleus over the full-time course of data collection) and a single TIFF image file outlining the ROI, and these are then combined to define the fluorescence intensity time series within the ROI (Figure 1).

Previously, the Q-FADD workflow accomplished this through a MATLAB notebook that utilized the BioFormats library^(13,22)^. In the Python workflow presented here, this is handled with the “image_analyzer.py” program, which uses the Python implementation of BioFormats along with microscope-specific libraries^(23)^. This program takes the single ROI TIFF file and the time-lapsed image stack as inputs, and it saves plain text files containing the accumulation data, a mask of the nuclear envelope, and a trace of the ROI to be utilized by the main qFADD.py program.

During this conversion, there are several corrections that must be applied to maximize the integrity of the accumulation data. First, continuous exposure to the fluorescence excitation laser can cause photobleaching of the fluorophores over the course of the experiment, and the effects of photobleaching must be deconvolved from accumulation and dissipation processes. Therefore, the program applies a scaling factor to the intensity measured at each frame under the assumption that the total number of fluorescent molecules in the nucleus should remain constant:

(1) $$I^{\prime }(t)=I(t)*s=I(t)*\frac{I_{o}}{I_{net\;}(t)},$$

where *I*′*(t)* is the scaled and outputted ROI intensity value at time *t*, *I(t)* is the ROI intensity value from the raw image at time *t*, *s* is the scaling factor, *I*_*o*_ is the frame-averaged total pixel intensities contained within the nucleus in a reference set of frames, and *I*_*net*_*(t)* is the sum of pixel intensities in the nucleus at time *t.* The number of frames to include in the reference set for *I*_*o*_ is declared by the user at runtime, and we elected to reference the first six frames in the example below, as they contained only images of the nucleus prior to the DNA damage event.

The second potential artifact within accumulation data may result from nuclei with lateral drift during image acquisition. In these samples, the nuclear region of DNA damage may migrate beyond the damage ROI expected by the camera, yielding artificially low accumulation counts in later stages of the time course. To correct this error, image_analyzer.py uses the scikit-image library^(24)^, where image translations are identified by determining the cross-correlation between the initial frame and all subsequent frames in Fourier space^(25)^. Notably, this provides the ability to correct for linear motion in the field of view but not for rotations of the nucleus, both in- and out-of-plane with the camera, or for deformation of the nuclear profile. As such, users should still be selective when identifying nuclei for probing and further analysis.

### 2.2. The nuclear diffusion model in Q-FADD

In the Q-FADD approach, fluorescence intensity accumulation is modeled using free diffusion in the two-dimensional space of the image slice^(13)^. The nuclear mask coordinates are used to determine the boundaries of motion, and movement within the nucleus is conducted on a grid, analogous to the pixel-level information of the acquisition camera. A large number of simulated molecules (here, 10,000) are initially placed randomly along the nuclear grid, and a subset of these molecules—the “mobile fraction” (*F*)—is selected to participate in the simulated diffusion. Control studies showed that using significantly fewer particles (1,000) is insufficient to generate reproducible results and using more sample points (16,000) yields no change in outcome beyond a negligible reduction in noise for the accumulation kinetics. For all the proteins we have tested and for those reported in numerous other publications (reviewed in Reference (9)), uniform distribution within the nucleus (at the resolution of the experiment) provides a good description of the proteins of interest. Physically, the mobile fraction parameter arises from the fact that some molecules within the nucleus will not move to sites of DNA damage for various reasons that include their tight binding to chromatin or that the amount of DNA damage is small compared with the total number of available proteins.

Molecules within the mobile fraction then undergo diffusion through a Monte Carlo scheme. For every iteration, each mobile particle is able to move either “left” or “right” with equal probability, and the magnitude of the motion is defined by a constant step size per iteration (*Δx*, in pixels or “grid points”). The same is true for the motion in the “up” and “down” directions. This results in motion described by

(2) $$D_{eff}=\frac{1}{2}\frac{{\left(\Delta x\;\cdot \;p\right)}^{2}}{\Delta t},$$

where *D*_*eff*_ is the effective diffusion coefficient, *Δx* is the step size in pixels per iteration, *p* is the pixel resolution, and *Δt* is the associated timestep with each iteration. We note that we chose this simplest definition of *D*_*eff*_ because it is sufficient to explain all the proteins we have tested (Occam’s razor), but that *D*_*eff*_ within the complicated milieu of the nucleus may include sub-diffusive processes or reaction-limited kinetics. From our experience, *Δt* should typically be on the order of 0.2 s per step, or about one order of magnitude higher resolution than the framerate of the camera. However, longer timesteps may be required to model slower diffusing particles, and the loss of sampling between experimental timepoints should be offset with an increased number of replicates per (*D*_*eff*_, *F*) pair. *D*_*eff*_ is used in place of the exact diffusion coefficient as the modeled—and experimentally observed—motion is a convolution of rapid binding and unbinding processes in addition to molecular diffusion.

During the Monte Carlo routine, boundary conditions are applied such that movement beyond the nuclear envelope is rejected. Furthermore, once a molecule has entered the simulated region of DNA damage (defined as the intersection between the ROI of the experiment and the nuclear mask), then it is considered to be trapped at the site of DNA damage. As such, the program is designed to model the accumulation kinetics of damage-response proteins, but it does not account for dissipation of proteins upon DNA repair or completion of signaling task. Users should therefore be careful interpreting results of proteins that dissipate very quickly according to the intensity time series.

The simulated trajectory is then converted to an intensity time series by counting the number of molecules contained within the ROI at a given iteration of the simulation. To allow for one-to-one comparison between the simulation and the experiment, both time series are normalized. In this way, *I*(*t*) defines the fold increase of fluorescence (or the number of simulated particles) from the initial frame, rather than the exact number of proteins/particles themselves. For the experimental data, normalization is achieved by dividing the ROI intensity of each frame by the average ROI intensity of the pre-damaged frames. In the simulated trajectories, this corresponds to dividing the number of particles in the ROI by the number of particles randomly initialized in the region prior to motion. The simulated intensity time series is then interpolated to the experimental timepoints, and fits to data are reported using two different quality-of-fit metrics: the *r*^2^ metric and the root-mean-squared deviation (RMSD) between the modeled accumulation time series and the experimentally observed fold increase of fluorescence intensity in the ROI. The RMSD metric is defined as

(3) $$RMSD=\sqrt{\frac{1}{N}\sum {\left(I_{qFADD}-I_{exp}\right)}^{2}},$$

where the sum is carried out over the *N* timepoints in the curve, and *I*_*qFADD*_ is the normalized fluorescence intensity at the interpolated timepoint associated with an experimental normalized intensity, *I*_*exp*_.

**Figure 2.**
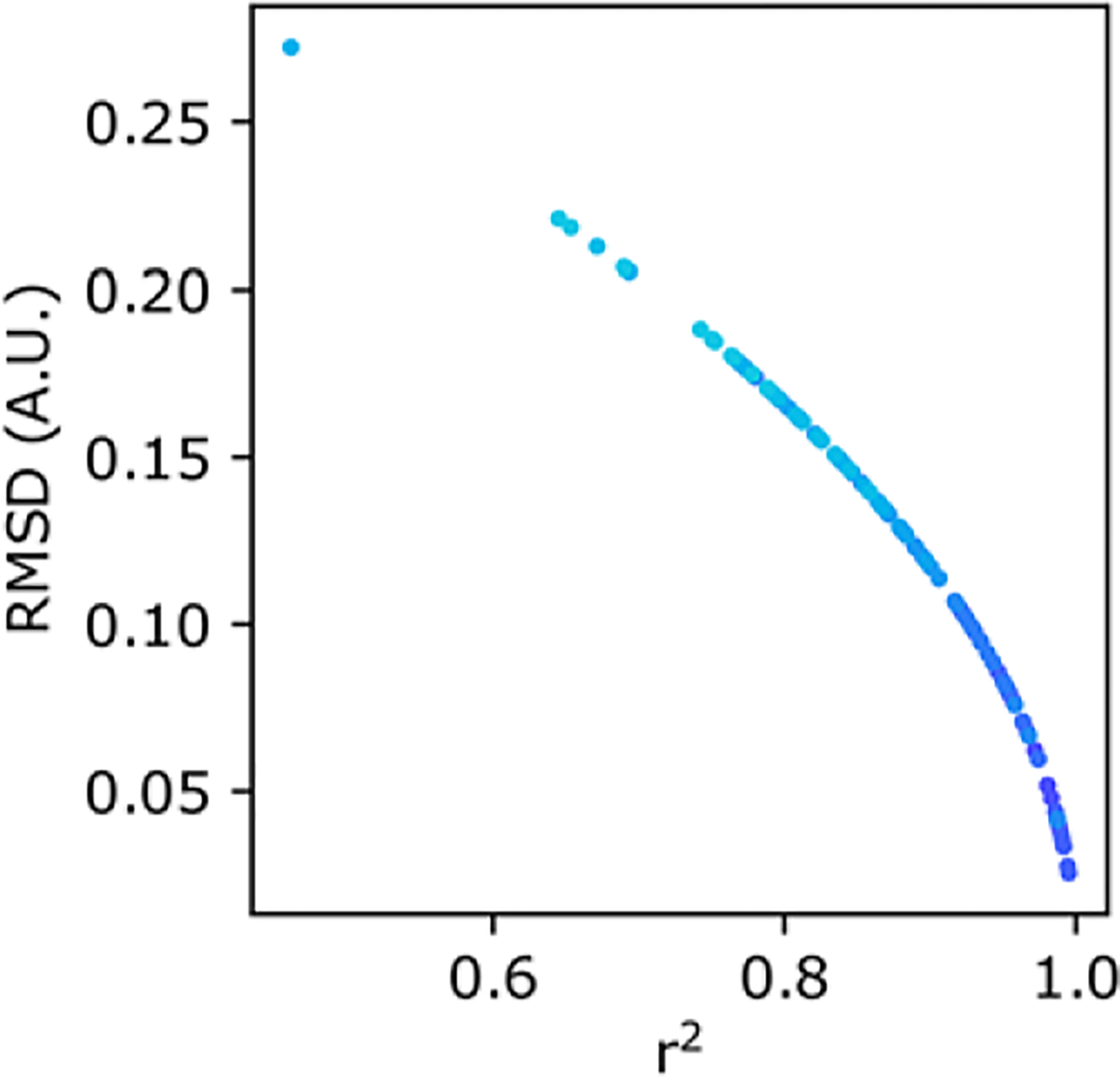
Scatterplots comparing the different goodness-of-fit metrics reported by qFADD.py. Scatterplot of *r^2^* versus root-mean-squared deviation (RMSD) values for individual diffusion models fit to the same nucleus. Each point is color-coded to match replicate simulations using identical (*D_eff_, F*) combinations (cumulative set of 11 replicas from 10 combinations shown here). While not linearly correlated, *r^2^* and RMSD are in perfect agreement for their ranking of each qFADD.py run. Nevertheless, the changing slope to a more vertical line near *r^2^* = 1.0 shows that the RMSD metric is better able to differentiate between models of similar high quality since small differences in *r^2^* correspond to larger differences in RMSD.

In our experience, both *r*^2^ and RMSD metrics agree on which parameter combination yields the best model. The benefit of using RMSD is that it is in the same units of normalized intensity and can better differentiate models of high-fit qualities (Figure 2). The RMSD value thereby tells the user an average discrepancy between the experiment and the model. In this way, RMSD values closer to zero represent high-quality fits and poor-fits diverge to large values. In contrast, *r*^2^ values ≈ 1.0 represent good fits, and because many users may be more familiar with this metric, we present both as outputs from the qFADD.py program and here within the manuscript.

### 2.3. Automated model fitting

The initial version of the Q-FADD algorithm provided the basic algorithm for accurately quantitating the accumulation of repair proteins to sites of DNA damage. However, it still required users to manually seek out individual combinations of *D*_*eff*_ and *F* through trial and error, which can be difficult, can require large investments of user time and effort, and benefits from prior experience. In the new qFADD.py workflow, the task of identifying the model of best fit has been largely removed from the user by utilizing a grid-search algorithm of the values *D*_*eff*_ and *F.* In this approach, users define their desired range and resolution of *D*_*eff*_ and *F* values, and the program evaluates the quality of fit for each combination of values. Computational efficiency of this search is enhanced by running tasks in parallel, where each processor is assigned a list of (*D*_*eff*_, *F*) parameter pairs to evaluate. Because Q-FADD is a sampling-based algorithm, multiple replicates are simulated per grid point to ensure that the fit qualities are statistically relevant and not the result of spurious random sampling. As a readout, users can decide between representing the model with either the replica trajectory with the median fit or as an averaged trajectory across all the replicas before fitting to the experiment. With an appropriate number of replicates (*n* > 10 replicates), the model qualities of median and averaged trajectories are typically found to be in good agreement with one another.

While grid-search algorithms will identify the single most likely combination of parameters to fit the experimental data, they also produce a variety of alternative models that adequately explain the results. As such, the qFADD.py program both prints the best-fitting model and stores the entire library of sampled parameters and their modeled goodness-of-fit values in an easy-to-read text file. In this way, users can explore the full catalog of potential models and supplement their intuition gained from orthogonal measurements, rather than fully replacing those results.

### 2.4. image_analyzer.py and qFADD.py parameters for example usage

To demonstrate how to use and interpret the qFADD.py workflow, we provide an example using data from a laser microirradiation experiment using mouse embryonic fibroblast (MEF) cells that overexpress the DNA damage signaling protein PARP1, tagged with the green fluorescent protein (GFP-PARP1). A full methods description for this experimental setup has been published^(13)^. Cell images were captured on a Nikon A1R laser scanning confocal microscope (Nikon, Tokyo, Japan). Accumulation of GFP-PARP1 was monitored using a 488-nm wavelength argon-ion laser, and DNA damage was introduced with a 405-nm diode laser focused at ~1.7 mW on a rectangular ROI for 1 s. Six frames were captured prior to DNA damage for the purpose of normalizing the accumulation time series, and the detector resolution was 86.77 nm per pixel. In total, we collected and analyzed data on 11 independent nuclei. One image stack is provided at the GitHub site as an example (example_files.zip), and a modest lateral drift is observed over the course of this collection (Supplemental Movies 1 and 2; available at the GitHub site). This causes the physically damaged region to move slightly beyond the ROI of the camera in the +*x* direction, meaning that this nucleus should have been potentially excluded unless drift correction was applied, depending on the decision of the user. More drastic motion was observed in a different image stack (Supplemental Movies 3 and 4; available at the GitHub site), and we present this individual nucleus to understand the importance of motion correction in the qFADD.py pipeline (Section 3.1). Presented results were generated using a local installation of qFADD.py.

First, the image stack and ROI TIF files were run through image_analyzer.py, where both protein accumulation and the nuclear mask were tracked by the fluorescently labeled protein channel (“EGFP”). Nuclear masking via the EGFP channel was selected because the protein was overexpressed and localized to the nucleus to provide a quasi-uniform outline of the nuclear boundary. Although it may seem intuitive to use the “DAPI” channel that marks DNA instead, this channel has regions of variable brightness that make it more difficult to generate a nuclear outline. Next, the *X*- and *Y*-limits of the ROI were, respectively, adjusted by −10 and 10 pixels to account for edge effects in the irradiated region, and drift correction was applied. The first six (pre-irradiated) frames were used to normalize the time series and correct for photobleaching. Then, the nuclear mask and ROI trace files, along with the accumulation time series, were used as input for the grid-based fitting of *D*_*eff*_ and *F* in qFADD.py grid. To sample *D*_*eff*_ values, the step size *Δx* ranged from 1 to 15 pixels per iteration with a grid point every integer value. To sample the mobile fraction, *F* values spanned from 100 to 1,000 parts per thousand (ppt) with a grid point every 100 ppt. Reported fits are the median model from each set of replicates, and 11 replicate trajectories were run per grid point. Each simulation was conducted at a timestep of 0.2 s per step, which yields a *D*_*eff*_ range of 0.02–4.2 μm^2^/s (see Equation (2)).

## 3. Results

### 3.1. Effects of motion correction

**Figure 3.**
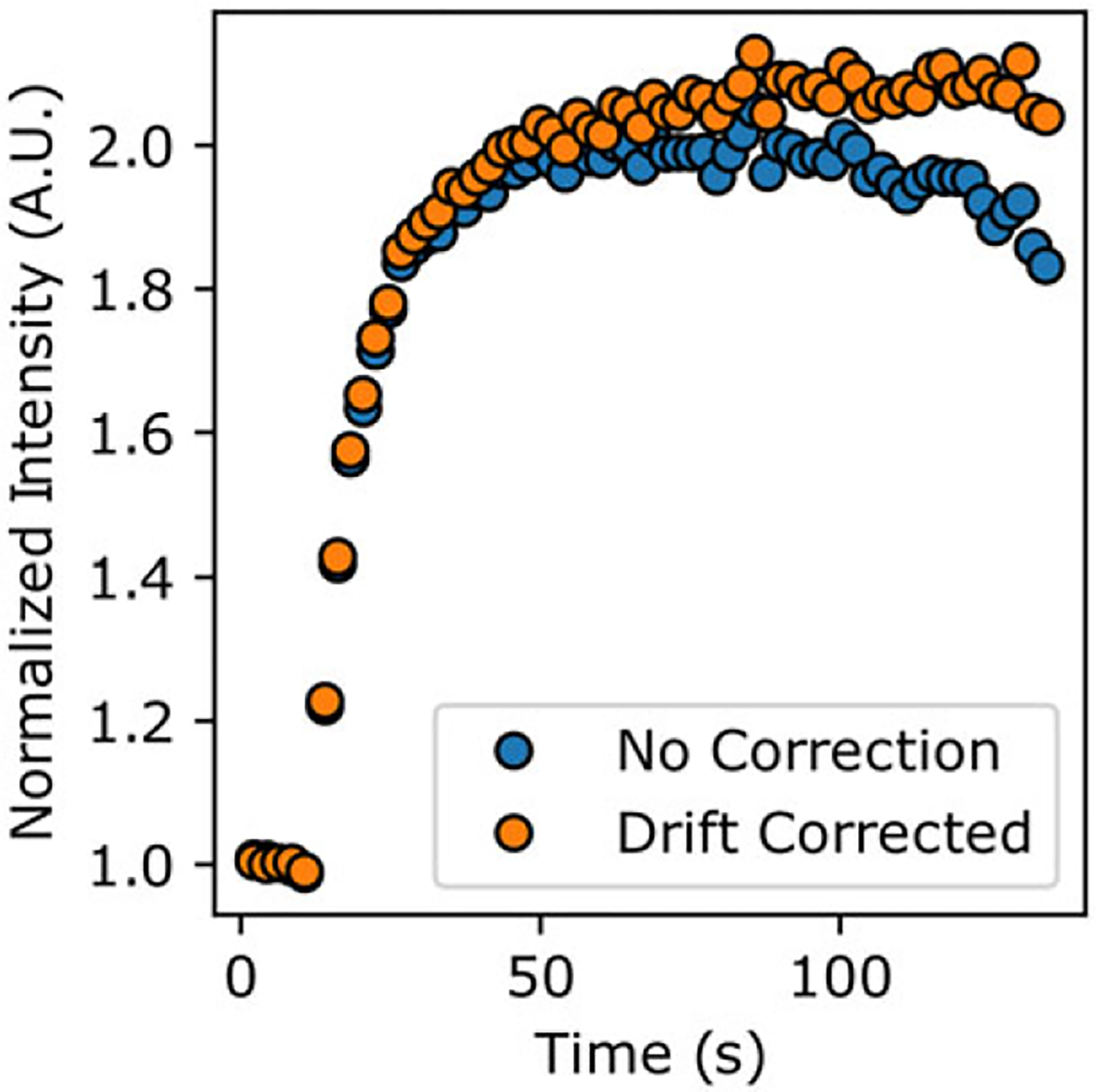
Comparison of accumulation time series for raw (blue) and drift-corrected (orange) processing. Notably, the curve extracted from the raw movies indicates a dissipation of tagged protein, but properly applying drift correction demonstrates that this is an artifact of the damage site exiting the region of interest. Moreover, non-corrected movies show less overall accumulation.

To probe the effects of neglecting motion correction on the measured accumulation time series and modeled diffusion behavior, we ran the image stack containing the nucleus with the largest lateral motion through image_analyzer.py with and without motion correction applied, while all other settings were held constant. Indeed, we find that there is a quantitative difference between the raw versus motion corrected time-series data (Figure 3). While the maximum accumulation amount between the two procedures differed by only 3.3%, the time series extracted from the raw image stack appears to show PARP1 dissipating from the ROI at late timepoints. With motion correction applied, there is no dissipation of PARP1 from the damage site in this timescale, which was visually confirmed by inspecting the images (Supplemental Movies 3 and 4). Additionally, the best-fit diffusion coefficients differ by nearly 15% (2.3 versus 2.7 μm^2^/s for the raw and motion-corrected data, respectively). This comparison demonstrates the importance of properly accounting for nuclear motion during protein accumulation, and as this is now implemented in the preprocessing that also accounts for photobleaching, it is readily accessible to the experimentalist.

### 3.2. Interpreting results from a single recruitment image stack

**Figure 4.**
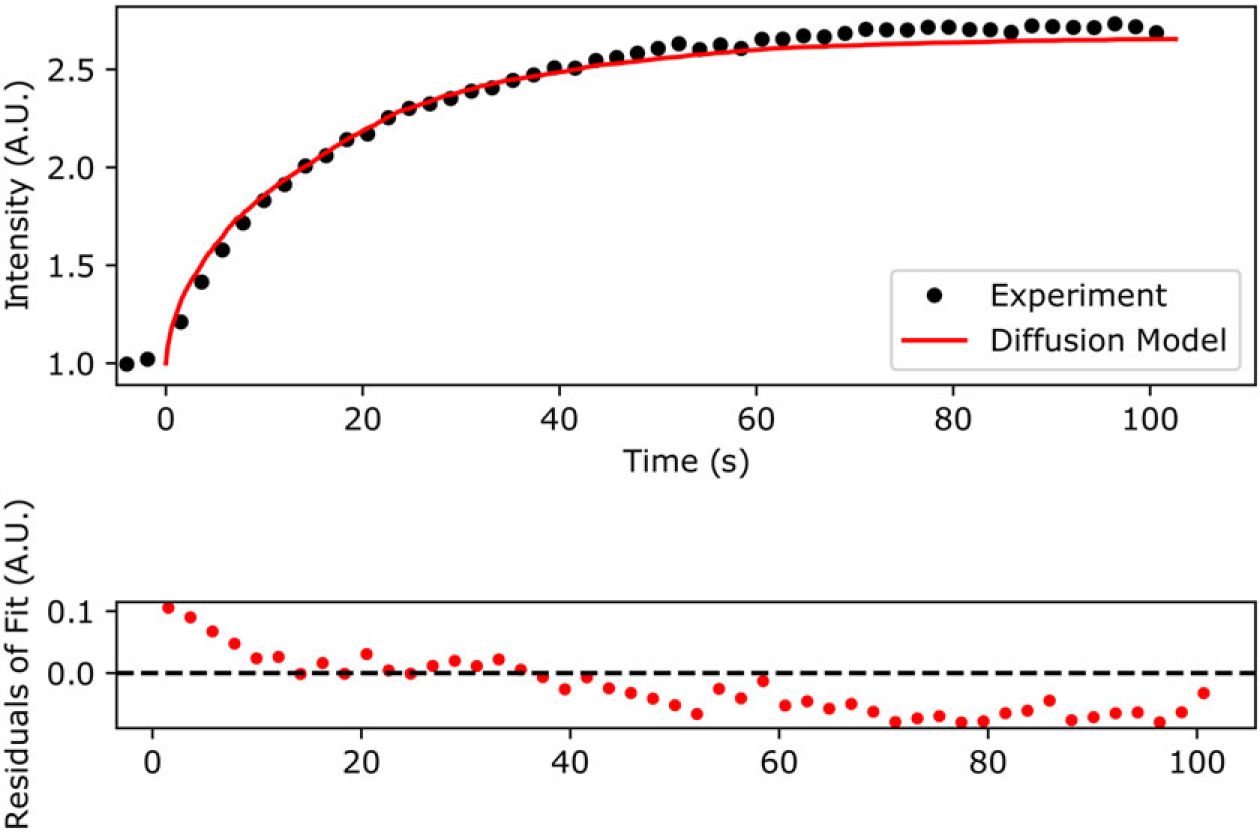
Comparison of the qFADD.py grid-search-identified “best-fit” model to the experimental accumulation time series for a single nucleus. (Top) Simulated accumulation via free diffusion (red line) versus the experimental profile (black dots). (Bottom) Residuals of the fit between the simulation and the experiment. Plotted data are the highest fit-quality median replicate from selected from all sampled median quality replicates in the grid-search space (11 replicates per combination of *D_eff_* and mobile fraction).

As an example of the new grid-search method, we inspect the qFADD.py modeling results for the nucleus containing a modest amount of lateral motion. This nucleus was selected due to its high-fit quality (Figure 4), the presence of several alternative models with appreciably high qualities of fit—which shows the selective power of the grid-search algorithm—and replicates of the same (*D*_*eff*_, *F*) combinations displayed wide ranges of model qualities, as discussed below.

**Figure 5.**
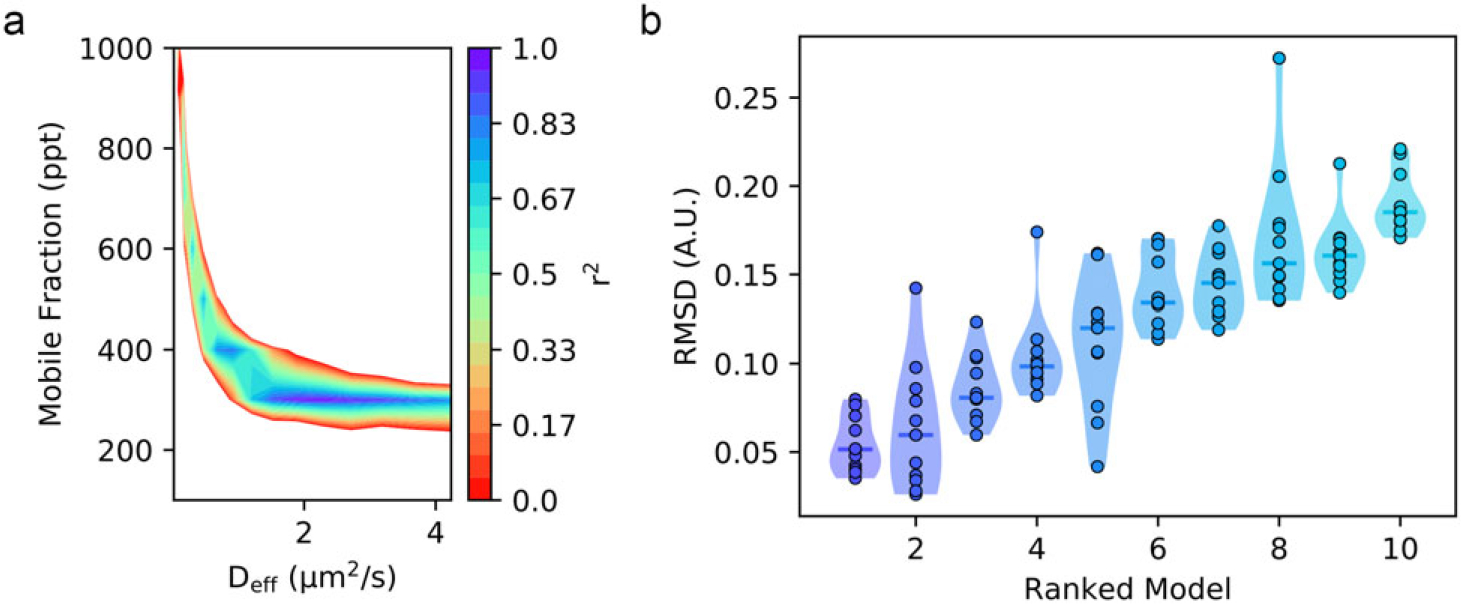
Comparisons of sampled model performances during the qFADD.py grid-search algorithm for a single nucleus. (a) Heatmap showing multiple local regions of relatively high (*r^2^* > 0.9) fit, showing that several different parameter sets may adequately describe the experimental profile for the accumulation at the region of interest (ROI) in a single nucleus. However, only the population centered around a mobile fraction of 300 ppt contains the models with *r^2^* values greater than 0.95. (b) Violin plots showing the root-mean-squared deviation distributions across the 11 independent replicates for the 10 best-performing (*D_eff_, F*) combinations on the same experimental dataset at the ROI in a single nucleus. Lower values correlate to simulated models that are more similar to the experimental profile. These plots show that because of the random sampling nature of the algorithm, some parameter combinations have spuriously high (or even low) fit qualities. Thus, by performing 10+ replicates, the median fit qualities (solid lines) provide a more robust ranking metric. The mean *D_eff_* and *F* from the best-ranked model for each nucleus are then averaged to determine the mean *D_eff_* and *F* for the population of other nuclei studied under the same conditions.

By inspecting the grid-search models of the motion-corrected accumulation time series from a single nucleus (Figure 5a), we find a range of potential models with a mobile fraction of 300 ppt that possess high-fit qualities. In this region, there are four models possessing *r*^2^ values greater than 0.9, and two of these models have *r*^2^ values greater than 0.97 (*D*_*eff*_ = 11 pix/step, 2.3 μm^2^/s, *r*^2^ = 0.9807, RMSD = 0.0515 and *D*_*eff*_ = 10 pix/step, 1.9 μm^2^/s, *r*^2^ = 0.9742, RMSD = 0.0596). We note that these top two models differ by only 1 pix/step. We delve further into the details of the 11 replicates sampled during the qFADD.py grid search to make several important points (Figure 5b). First, we note that each of the models has a range of RMSD values, and that by performing the simulations only once, one may not end up with the best solution. This finding emphasizes the value of performing replicate simulations in a procedure that is based on sampling random movement of particles. Second, for the best model identified during the grid search (*D*_*eff*_ = 2.3 μm^2^/s, *F* = 300 ppt; left-most model in Figure 5b), we find that the *r*^2^ values range from 0.9572 to 0.9910 (RMSD range of 0.0767–0.0351). This narrow range of the fitting parameters *r*^2^ and RMSD emphasizes the robustness of this fit to random error in the model. Third, the parameter combination that ranked second (*D*_*eff*_ = 1.9 μm^2^/s, *F* = 300 ppt) contains both the best single replicate in the entire search and the worst-fit replicate (*r*^2^ = 0.8529, RMSD = 0.0788), and this model is notably worse than any in the best-fit model (note extended neck in the violin plot). In summary, the grid search employed within qFADD.py efficiently protects users from bias from single replicates and the automated search robustly identifies the model of optimum fit by scanning a wide range of values and running multiple replicates per parameter combination.

### 3.3. Combining results from multiple nuclei and comparing between populations

**Figure 6.**
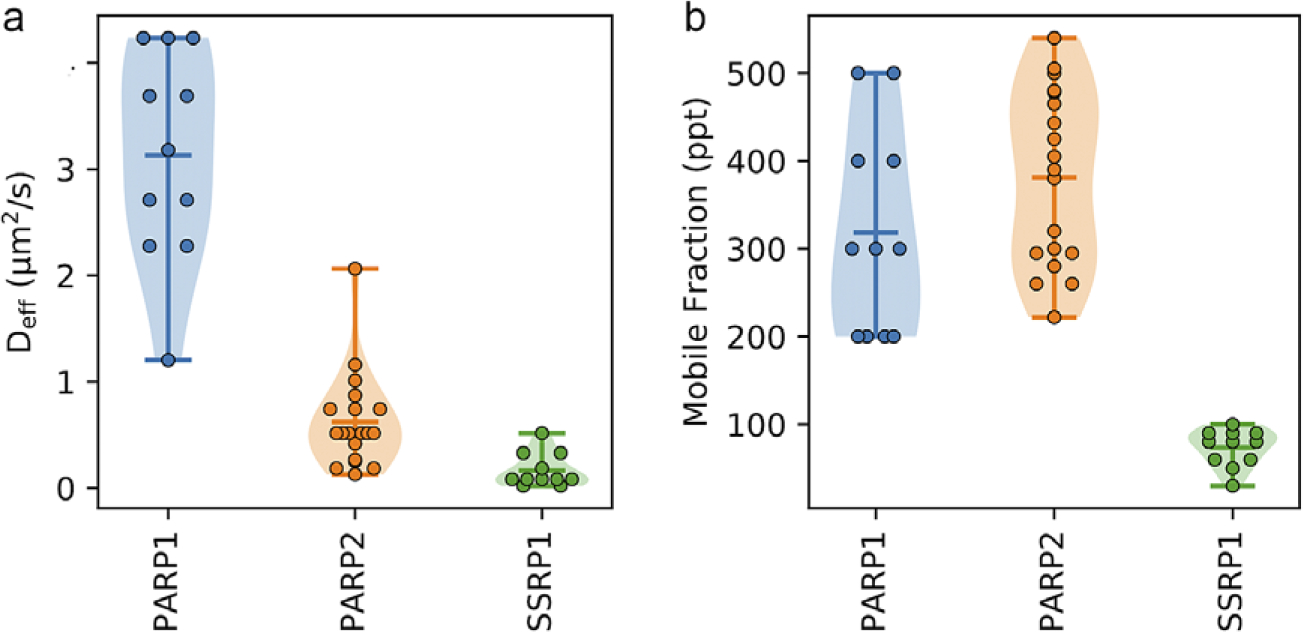
Examples of the comparison violin plots that can be generated with the qfadd_distribution.py program. (a) Plot of *D_eff_* generated when comparing between multiple datasets. The PARP1 distribution is representative of the 11 cells sampled in the work of this manuscript. The PARP2 distribution is extracted from our previous study on PARP2 dynamics^(13)^. The SSRP1 dataset demonstrates data from the SSRP1 subunit of FACT (11 cells). As previously observed, the *D_eff_* for PARP1 was significantly faster than that of PARP2 (*p*-value < .001), and both PARP1 and PARP2 are significantly faster than SSRP1 (*p*-value < .001). (b) Comparison plot of mobile fractions (*F*), which demonstrates no significant difference between PARP1 and PARP2, but a significant difference for SSRP1 (*p*-value < .001).

Above, we demonstrated the functionality of the qFADD.py grid-search approach for a single nucleus. However, one of the strengths of the Q-FADD approach is the ability to identify a distribution of *D*_*eff*_ and *F* values across a large number of nuclei, correctly accounting for their differing shapes and sizes. To facilitate the analysis of multiple nuclei, both the qFADD.py and image_analyzer.py programs can be run in a “batch” mode. To use the “batch” processing mode, every nucleus in the dataset must have identical parameters such as number of normalization frames prior to irradiation, the time of the irradiation event, and a sufficient timescale such that maximum accumulation was reached. To facilitate comparisons between different conditions or different proteins, we provide the “qfadd_distribution.py” program, which generates violin plots from a set of Q-FADD results with multiple nuclei. In an example application, we collected and analyzed microirradiation data for 11 and 19 different nuclei for PARP1 and PARP2, respectively, and plotted the range of diffusion coefficients and mobile fraction for the 11 best-fit models for each of these proteins as identified by the grid search in qFADD.py (Figure 6). This comparison shows that there is a significant difference between the modeled diffusion rates in the two datasets (*D*_*eff*_ = 3.1 ± 0.3 vs. 0.62 ± 0.10 μm^2^/s, *p*-value < .001), but no significant difference in the mobile fraction comparison (*F* = 318 ± 34 ppt vs. 381 ± 22 ppt, *p*-value = .13). In general, we find that *D*_*eff*_ and *F* are determined with good reproducibility and very similar percentage variations in the standard error of the mean of all the determined values (6–14%), depending of course on the protein and the sample size. These data agree with previous observations that PARP1 reaches sites of DNA damage significantly more quickly than PARP2, as previously reported^(5,13)^ and demonstrates the value of this program for multiple datasets.

## 4. Discussion

The qFADD.py analysis pipeline is an open-source Python implementation of the Q-FADD method^(13)^ with updated image processing, diffusion-fitting procedures, and additional analysis tools. The software is available as a stand-alone program for local installation (https://github.com/Luger-Lab/Q-FADD). By providing these tools, researchers can readily analyze their own microirradiation data, while labs with cluster access can make efficient use of the parallelized grid search algorithm on their local resources.

The qFADD.py pipeline has introduced several significant improvements over the original implementation. One major improvement is the addition of nuclear tracking to the image processing workflow, which corrects for lateral drift and allows researchers to avoid discarding valuable image stacks, as well as safeguarding them from interpreting nuclear drift as the dissipation of fluorescently labeled proteins from the damage site. This allows researchers to maximize the number of usable datasets in their analysis and improves statistical rigor when comparing accumulation kinetics between cell conditions or proteins of interest. We have shown here that modeling accumulation kinetics from non-corrected image stacks can lead to quantifiable differences in predicted values. Furthermore, the time-series data extracted by the image_analyzer.py program is not limited to use only in the qFADD.py program, but it can also be used to extract dissipation time series for modeling parameters such as off-rate kinetics. We note that the corrections applied in image_analyzer.py only corrects for lateral drift and cannot account for cells that undergo rotations either in- or out-of-plane with the camera.

While motion correction during image processing extends the number of nuclei that can be included in final datasets, careful selection of nuclei should still be performed while collecting microscope data. Nuclei with blebs may pose issues for accurately tracing the nuclear envelope, and these nuclei may subsequently yield *D*_*eff*_ and mobile fraction combinations that can satisfy the experimental curve but with an improper nuclear geometry, thereby representing a false-positive result. Additionally, users should carefully control microscope temperature and humidity to maintain healthy cells over the course of collections.

The second key improvement to the original Q-FADD algorithm is the utilization of the automated grid search to identify the best-fit model from a collection of potential models. With the use of multiple replicates per parameter set, this method determines the best-fit model in a statistically robust way, whereas a user’s trial-and-error search may be prone to identifying spuriously high-fit models. On the other hand, the grid-search method requires a balance of resolution and computational efficiency, as searching out a wide range of mobile fractions on the single ppt level is exceptionally demanding. Instead, users should first identify general regions of high model fidelity utilizing a coarse grid step (such as 100 ppt), and then focus in on the boundaries of said regions at higher resolution (such as 10 or 25 ppt) to extract more precise values of this metric. However, the resolution limit of the mobile fraction parameter is largely dependent on the recruitment speed, protein expression levels, and fluorescent labeling efficiency, so users should be careful to avoid overfitting the mobile fraction value with single ppt resolution in systems where 10–50 ppt will suffice.

In summary, the qFADD.py pipeline, with its automated workflow, provides researchers with a straightforward way to model the kinetics of proteins that operate in a variety of DNA repair pathways. In combination with multi-photon approaches, this method allows the opportunity to simultaneously probe the dynamics of several proteins within the same nucleus, rather than relying on separate image stacks from different nuclei.

## Supplementary Material

cell004-raw

cell001-drift

cell003-raw

cell001-raw

cell002-raw

cell004-drift

cell-003-drift

cell002-drift

## Data Availability

All raw data and the code described in this manuscript are available at https://github.com/Luger-Lab/Q-FADD.

## Impact Statement

Live-cell microscopy and data analysis have been particularly impactful in the study of complex DNA repair processes. Over recent years, innovations in microscopy have allowed for imaging of processes occurring inside cells or even inside nuclei. These improvements have been particularly impactful in the study of DNA repair processes. For example, the method known as laser microirradiation has become a popular in vivo technique to study how fluorescently tagged DNA repair proteins accumulate, reside, and then dissipate from sites of DNA damage induced by laser damage of the DNA in live cells. Unfortunately, analysis of laser microirradiation data is often performed incorrectly or incompletely, thus precluding comparisons between different studies or with other methods. Because the correct and quantitative analysis of biological processes is essential for a more complete understanding of life processes, we previously developed the “quantitation of fluorescence accumulation after DNA damage” method, which allows for the determination of true diffusion coefficients and thus enables comparison with other methods. Here, we describe the automated and improved implementation known as qFADD.py, which allows any biologist to accurately quantitate their data from laser microirradiation.
